# New aspects of microbial vitamin K2 production by expanding the product spectrum

**DOI:** 10.1186/s12934-021-01574-7

**Published:** 2021-04-13

**Authors:** Zimeng Zhang, Linxia Liu, Chuan Liu, Yumei Sun, Dawei Zhang

**Affiliations:** 1 School of Biological Engineering, Dalian Polytechnic University, Dalian, 116034 China; 2 Key Laboratory of Systems Microbial Biotechnology, Chinese Academy of Sciences, Tianjin, 300308 China; 3 Tianjin Institute of Industrial Biotechnology, Chinese Academy of Sciences, Tianjin, 300308 China; 4 National Technology Innovation Center of Synthetic Biology, Tianjin, 300308 China; 5 University of Chinese Academy of Sciences, Beijing, 100049 China

**Keywords:** Vitamin K2, Biosynthetic pathway, Pathway engineering, Product spectrum

## Abstract

Vitamin K2 (menaquinone, MK) is an essential lipid-soluble vitamin with critical roles in blood coagulation and bone metabolism. Chemically, the term vitamin K2 encompasses a group of small molecules that contain a common naphthoquinone head group and a polyisoprenyl side chain of variable length. Among them, menaquinone-7 (MK-7) is the most potent form. Here, the biosynthetic pathways of vitamin K2 and different types of MK produced by microorganisms are briefly introduced. Further, we provide a new aspect of MK-7 production, which shares a common naphthoquinone ring and polyisoprene biosynthesis pathway, by analyzing strategies for expanding the product spectrum. We review the findings of metabolic engineering strategies targeting the shikimate pathway, polyisoprene pathway, and menaquinone pathway, as well as membrane engineering, which provide comprehensive insights for enhancing the yield of MK-7. Finally, the current limitations and perspectives of microbial menaquinone production are also discussed. This article provides in-depth information on metabolic engineering strategies for vitamin K2 production by expanding the product spectrum.

## Introduction

Dr. Henrik Dam discovered vitamin K in the 1930s and shared the 1943 Nobel Prize in medicine with Edward Doisy for their work on this fat-soluble bioactive compound [1–3]. Vitamin K serves as a cofactor for γ-glutamyl carboxylase (GGCX), which converts glutamic acid residues of vitamin K-dependent proteins (VKDPs) into γ-carboxyglutamic acid (Gla). There are more than 15 types of VKDPs, including coagulation factors II (prothrombin), VII, IX, and X, as well as the anti-coagulation factors C, protein S, and osteocalcin [4–7]. Additionally, optimal vitamin K status is strongly associated with various health benefits, such as preventing or alleviating cardiovascular disease, osteoporosis, osteoarthritis, cancer, inflammatory diseases, diabetes, chronic kidney disease, immune disorders, and Alzheimer’s disease [4, 7–10]. Perhaps unsurprisingly, vitamin K deficiency may also influence the mortality and morbidity of COVID-19 patients [11].

**Fig. 1:**
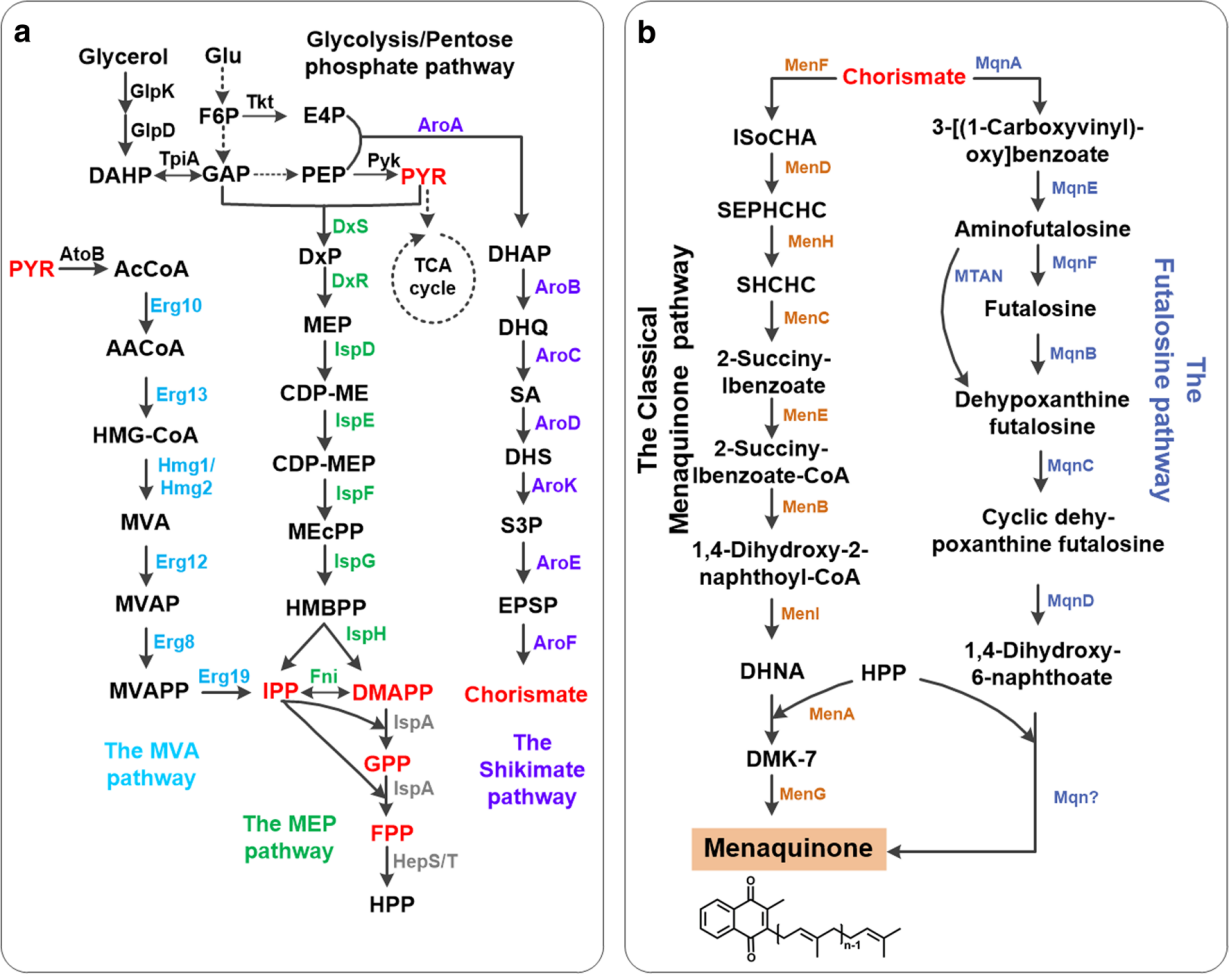
The biosynthesis pathways of menaquinone in isoforms from *Bacillus subtilis*, *Saccharomyces cerevisiae* and *S. coelicolor* [26, 37, 41]. Menaquinone biosynthesis is a complex process involving multiple metabolic pathways, such as glycolysis, the pentose phosphate pathway, the shikimate pathway, the MEP or MVA pathway, as well as the classical MK pathway or futalosine pathway. **a** The important intermediates chorismate and polyisoprene are synthesized from the precursors G3P, pyruvate and E4P. **b** The classical pathway and the futalosine pathway for the biosynthesis of menaquinone. Enzymes are displayed in different colors in different pathways. Red typeface indicates several important intermediate metabolites

The term vitamin K denotes a series of fat-soluble compounds that contain a 2-methyl-1,4-naphthoqumone moiety as the basic skeleton and an isoprenoid chain at the 3-position [12]. Based on the side-chain structure, vitamin K isoforms are categorized as vitamin K1 (phylloquinone, PK), vitamin K2 (menaquinone-n, MK-n) and vitamin K3 (menadione, MD) (Fig. 1) [13–15]. Vitamin K3 is a synthetic product without a side chain, while vitamin K1 and K2 occur naturally. Vitamin K1 has an aliphatic side chain and is predominately found in various leafy green vegetables, fruits, and plant oils [13, 16]. Vitamin K2 contains an unsaturated aliphatic side chain with a variable number (n) of 4 to 13 isoprene units and is referred to as MK-n [17]. Vitamin K1 is currently produced via chemical synthesis, and there is little evidence of successful attempts to reduce or eliminate the utilization of toxic chemicals in this process by applying enzymes [13, 16]. However, consumers are increasingly favoring “natural” products like vitamin K2 fermented by Generally Recognized as Safe (GRAS) bacteria [18, 19]. Thus, this review focuses on strategies for industrial vitamin K2 biosynthesis.

Among vitamin K2 homologues, MK-4 is the most common form in animals, with the widest range of physiological activities [6]. The long-chain MK isoforms, such as MK-7, are found in fermented foods or produced by bacteria. The traditional Japanese food natto, which is made from soybeans fermented by *Bacillus subtilis natto,* contains MK-7 at a very high concentration [1]. MK-4 and MK-7 are allowed in the United States as nutritional supplements for bone health [20]. However, the administration of MK-4 is not reflected in an increased serum concentration [6]. By contrast, MK-7 is absorbed efficiently, which is reflected in increased serum MK-7 levels up to several days, thereby contributing to vitamin K status [6, 21]. US Pharmacopeia monographs have been developed to establish quality standards for menaquinone-7 as a dietary ingredient at typically recommended levels [22]. Although, there are *cis*, *trans*, and *cis*/*trans* isomers of MK-7, only the all-*trans* form is biologically active [21]. It can be produced naturally through fermentation, as in the case of natto, or by organic synthesis. In the process of organic synthesis of MK-7, *cis*/*trans* isomers can be produced at the same time. Thus, the potential for the different isomeric forms of MK-7 to have different cytotoxic or toxic properties is an important consideration [21, 23]. Naturally derived MK-7 is produced via fermentation of generally recognized as safe (GRAS) bacterial strains such as *B. subtilis natto*, and is therefore considered to be nontoxic [2]. Thus, enhancing the production of vitamin K2 by environmentally friendly fermentation has been extensively studied.

The aim of this review is to offer new insights for improving microbial factories for the production of vitamin K2, with a particular focus on pathway engineering and membrane engineering by expanding the product spectrum. The bacterial MK biosynthesis pathway and the producing microorganism that synthesize different dominant MK isoforms (MK-4, MK-6, MK-7, MK-8, etc.) are briefly discussed. Finally, the limitations of microbial menaquinone production are also discussed.

### Biosynthetic pathway of vitamin K2

The selection of high-yield strains is based on the biosynthesis pathway of vitamin K2 in prokaryotes. In 1982, Bentley et al. [24] initially clarified the mechanism of vitamin K2 biosynthesis in bacteria and reviewed the detailed roles of shikimate, 2-succinylbenzoate, 1,4-dihydroxy-2-naphthoate, and other intermediates. They also described individual reactions in the pathway from the perspective of chemical mechanisms and enzymological characteristics [24]. The chemical structure of vitamin K2 includes a naphthoquinone ring and an isoprene side chain [25].

The naphthoquinone ring can be synthesized via the classical MK pathway or the futalosine pathway (Fig. 1) [26–28]. The classical MK pathway has been known for decades and is found in almost all aerobic or facultatively anaerobic prokaryotes. By contrast, the futalosine-dependent pathway was first discovered and validated in *S. coelicolor* A3(2) in 2008, and is found in a broader taxonomic range of organisms, including also anaerobic microorganisms [29–31]. Both the classical MK pathway and the futalosine pathway diverge at chorismate and re-converge after the formation of menaquinone [32]. Therefore, chorismate is the key intermediate for the biosynthesis of the menaquinone ring. Chorismate is synthesized via the shikimate pathway from two precursors: E4P, which is an intermediate product of the pentose phosphate pathway, and phosphoenolpyruvate (PEP), an intermediate product of the glycolysis pathway or glycerol metabolism (Fig. 1a) [33, 34]. In the first step of the classical MK pathway, MenF converts chorismate into isochorismate, which is further converted by six enzymes encoded by the *menDHCEB* operon to form 1,4-dihydroxy-2-naphthoate (Fig. 1b) [17, 26]. In the first step of the futalosine pathway, MqnA catalyzes the dehydration of chorismate to generate 3-[(1-carboxyvinyl) oxy]-benzoic acid, which is then converted into 1,4-dihydroxy-6-naphthoate via three enzymatic reactions catalyzed by MqnBCD (Fig. 1b) [31, 35, 36].

Secondly, the isoprene side chain is generally produced from two simple five-carbon units, isopentenyl diphosphate (IPP) and dimethylallyl diphosphate (DMAPP), either via the 2-C-methyl-d-erythritol-4-phosphate (MEP) pathway or the mevalonate (MVA) pathway [37, 38]. The MVA pathway is present in most eukaryotes, archaea, some bacteria, as well as cytosol and mitochondria of plants, while most bacteria, chloroplasts, and apicomplexan parasites synthesize IPP and DMAPP through the MEP pathway [37, 39]. The MEP pathway consists of eight enzyme-catalyzed reactions involved in the formation of IPP and DMAPP. Glyceraldehyde 3-phosphate (G3P) and pyruvate from the glycolysis pathway or glycerol metabolism are converted by 1-deoxy-d-xylulose-5-phosphate synthase (Dxs) to form 1-deoxy-d-xylulose-5-phosphate (DXP), which enters the MEP pathway. Then, DXP can be converted into MEP by 1-deoxy-d-xylulose 5-phosphate reductoisomerase (Dxr). IPP and DMAPP are produced by a pathway containing five enzymes, called IspDEFGH, but DMAPP can also be converted into IPP by isopentenyl-diphosphate delta-isomerase (Fni) (Fig. 1a) [40]. The MEP pathway starts with the condensation of G3P and pyruvate, while the MVA pathway uses acetyl‐CoA as a substrate. Acetyl-CoA is converted into acetoacetyl-CoA by acetyl-CoA acetyltransferase [41]. Then, the five enzymes Erg13, Hmg1/Hmg2, Erg12, Erg8, and Erg19 convert acetoacetyl-CoA to IPP. Finally, IPP undergoes a two-step catalytic reaction to form the isoprene side chain-polyprenyl-PP (C40) [41]. These two pathways have been widely used in studies of isoprene biosynthesis.

Finally, the membrane-bound enzymes polyprenyltransferase (MenA) and methyltransferase (MenG) combine the menaquinone ring and isoprene side chain to synthesize vitamin K2 [40].

### Different isoforms of vitamin K2 produced by various bacteria

In the Western diet, cheese and curd produced with *Lactococcus lactis* are the most important sources of vitamin K2. Bøe and Holo [42] co-overexpressed *mvk*, *preA*, and *menA* to increase the vitamin K2 yield (mainly MK-3, MK-7, and MK-9) (Table 1). When milk was fermented using the modified K2-overproducing strains, the vitamin K2 yield was effectively increased threefold compared to the wild type, which provides a foundation for the development of strains to ferment food with increased functional value [42].

**Table 1:** Metabolic engineering for the production of vitamin K2

Vitamin K2	Host	Pathway engineering	Strategies	Media	Titer	References
MK-7 and MK-9	*L. lactis*	The MVA pathway, the polyprenyl pathway and the MK pathway	Overexpression of *mvk*, *preA*, and *menA*	M17	680 nmol/L	[42]
MK-4	*B. subtilis*	The MVA and MEP pathway, the polyprenyl pathway and the MK pathway	Overexpression of *menA*, *menG*, *crtE*, *dxs*, *dxr*, *ispD*-*F*, *mvaK1*, *mvaK2*, *mvaD*, *mvaS*, *mvaA*	Fermentation medium	120.1 ± 0.6 mg/L	[44]
MK-4	*P. pastoris*	The polyprenyl pathway	Expression of *hsUBIAD1*	BMMY	0.24 mg/g DCW	[45]
MK-7	*B. subtilis natto*	–	Optimization of growth parameters	Fermentation medium	12.09 mg/L	[55]
MK-8	*E. coli*	The SA pathway, the ubiquinone-8 pathway and the MK pathway	*ubiCA* deletion, overexpression of *menA* and *menD*	LGN	290 μg/g WCW	[46]
MK-7	*E. coli*	The SA pathway	Overexpression *aroA*, *aroK* and the feedback inhibition-resistant *aroG*^fbr^		19.1 mg/L	[64]
MK-7	*B. subtilis*	The MEP pathway and the MK pathway	The expression of *menA*-*dxs*-*dxr*-*idi* cassette	SYG	50 mg/L	[75]
MK-7	*E. coli*	The polyprenyl pathway and the MVA pathway	Overexpression of *hepPPS* and lower expression of *mvaE*, *mvaS* and *mvK*	Minimal medium added glucose	2.3 μM	[77]

*DCW* dry cell weight, *WCW* wet cell weight

MK-4 is found in small amounts in animal products, such as eggs and meat, and it is also the major form (> 90%) of vitamin K found in animal tissues [9, 43]. MK-4 is mainly produced by microbial fermentation, which only produces the all-trans configuration, while the chemical synthesis of MK-4 remains a challeng [23]. Yuan et al. [44] improved the synthesis efficiency of MK-4 by combinatorial pathway engineering involving four modules, a MK-4 biosynthesis module (overexpression of *menA*, *menG*, and *crtE*), MEP module (knockout of *hepT*; overexpression of *dxs*, *dxr*, and *ispD*-*ispF*), MVA isoprenoid module (heterogeneous expression of *mvaK1*, *mvaK2*, *mvaD*, *mvaS*, and *mvaA*), and menaquinone module (overexpression of *menA* and *menG*). The MK-4 of resulting engineered *B. subtilis* increased to 120.1 mg/L in shake flasks (Table 1) [44]. Another example is in the methylotrophic yeast *Pichia pastoris*, in which a novel synthetic pathway for the production of MK-4 was introduced via heterologous expression of UbiA prenyltransferase containing 1 (HsUBIAD1). After optimizing the expression conditions, the yield increased 4.37 times compared with that under the initial conditions (Table 1) [45].

*Escherichia coli* is a facultatively anaerobic bacterium that produces benzoquinone-type ubiquinone-8 (Q-8) under aerobic conditions and mainly synthesizes naphthoquinone-type menaquinone-8 (MK-8) under anaerobic conditions. Kong and Lee [46] enhanced the MK-8 content by modulating two precursors pools and blocking the competing synthesis of Q-8. Further, overexpression of *menA* and *menD* increased the MK-8 yield fivefold compared with the wild-type (Table 1) [46].

Some enterobacteria such as *Eubacterium lentum*, *Veillonella, Enterobacterium*, and *Bacteroides* species can produce MK-6, MK7, MK-8, MK-10, and MK-11, but the exact mechanisms remain underexplored [47, 48].

The MK-7 long-chain isoform is an essential nutrient for humans because it is the most efficiently absorbed form of vitamin K and has a long half-life of 68 h compared with only 1–2 h for K1 [6, 49, 50]. *B. subtilis natto* is the main host for the industrial production of vitamin K2 [51]. It was also shown to produce a range of vitamin K2 isoforms, from MK-4 to MK-8, but MK-7 accounts for more than 90% of the total [52]. *B. subtilis natto* was originally used in the fermentation of natto, a traditional Japanese food, which is an excellent source of MK-7 [53, 54]. Optimization of *B. subtilis natto* growth parameters including temperature, pH, and agitation in a glycerol-based medium increased the MK-7 concentration to 12.09 mg/L in a biofilm reactor, without genetic modification [55]. Next, the review will focus on the metabolic engineering of microbes for the production of MK-7.

### Metabolic engineering of MK-7 production from the aspects of pathways and the product spectrum

In the pathways described above, the most important intermediates for vitamin K2 biosynthesis are chorismate and isoprene. Chorismate enters the classical MK pathway or the futalosine pathway to form the naphthoquinone head-group (Fig. 1) [40]. The polyisoprene tail is produced from two, five-carbon (C5) universal precursors: IPP and DMAPP through the MEP pathway or the MVA pathway [41]. Then, the polyisoprene tail is ligated with the naphthoquinone head by MenA to form demethylmenaquinone (DMK) and MK-7 is synthesized via the methylation of DMK [40]. Therefore, the key points of the pathways which may be the rate-limiting steps of vitamin K2 biosynthesis need to be discussed in depth. Undoubtedly, precursors for vitamin K2 biosynthesis also participate in the biosynthesis of various other chemicals, including some well-studied ones, such as terpenoids and aromatic acids. Therefore, this review not only discusses the unique metabolic engineering strategies for the production of vitamin K2 but also offers a perspective of engineering methods for other products that share the same precursors or pathways, thereby providing ideas for future research on vitamin K2.

### Metabolic engineering of the shikimate pathway to improve chorismate production

**Fig. 2:**
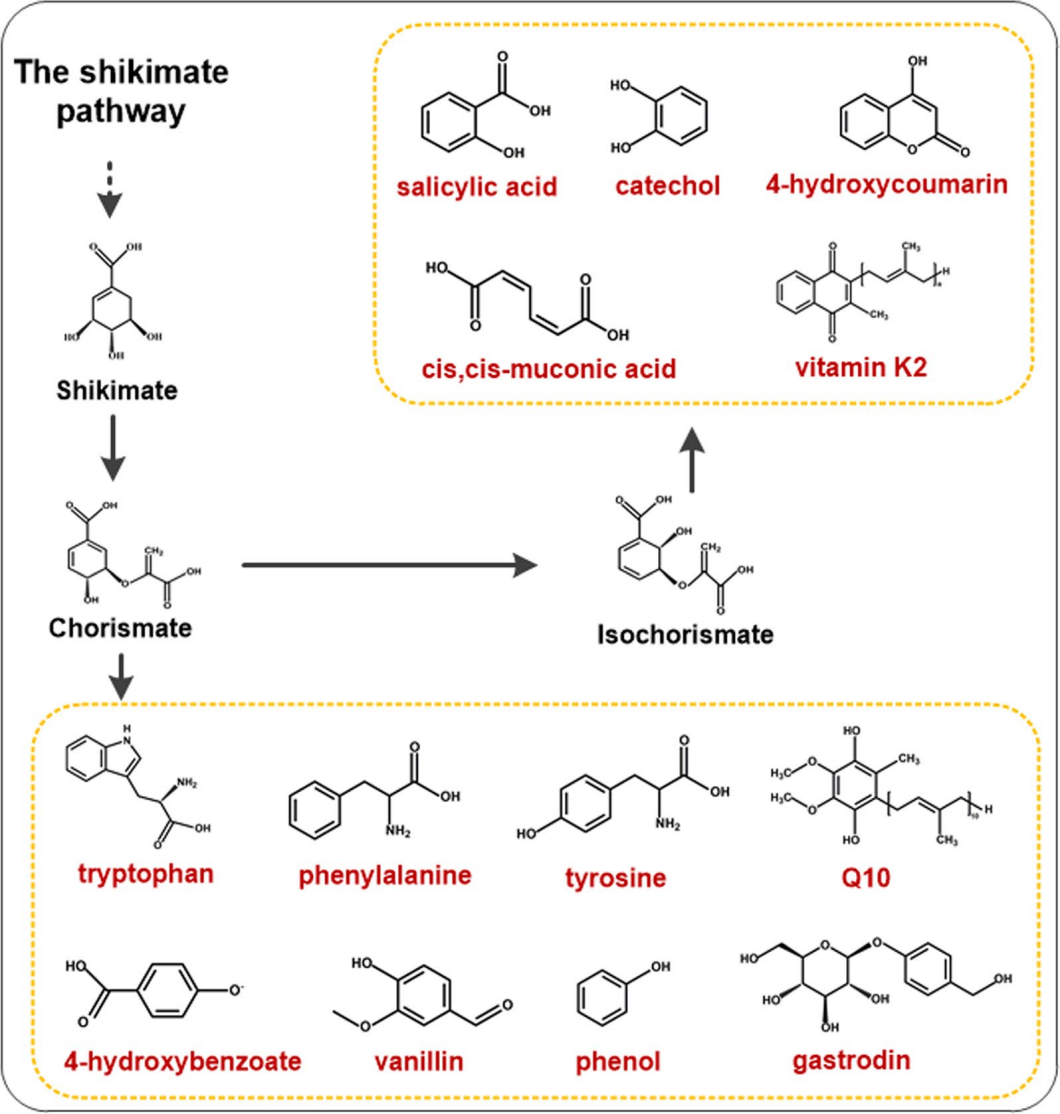
The shikimate pathway and heterologous products derived from chorismate and isochorismate. Red typeface indicates the product spectrum derived from chorismate or isochorismate

The shikimate pathway connects the central carbon metabolism with the biosynthesis of chorismate, which is a key precursor for the production of aromatic amino acids and a large number of other aromatic compounds in plants and microorganisms, including vitamin K2 (Fig. 2) [56–58]. In *E. coli*, three different 3-deoxy-d-arabino-heptulosonate-7-phosphate synthases (DAHPS) isoenzymes encoded by the AroGFH genes contribute to the total DAHPS activity and are subject to allosteric control by l-phenylalanine, l-tyrosine, and l-tryptophan, respectively [59–62]. Through structural analysis of mutant enzymes that are not sensitive to feedback, certain specific amino acid residues involved in the allosteric site have been identified, and feedback resistant (fbr) variants of AroG and AroF have been developed [59, 62]. Hence, elimination of feedback inhibition of the key enzymes obtained by introducing site-directed mutations is usually the first and most important step for the construction of a high-producing strain.

In a study of modular pathway engineering to promote MK-7 production, Yang et al. [63] found that overexpression of AroADE in *B. subtilis* inhibited the biosynthesis of MK-7, but the transcriptional levels of these genes were significantly increased (> 300-fold). It was suggested that increased production of aromatic amino acids resulted in feedback inhibition of the shikimate pathway, and ultimately inhibited the production of MK-7 [63]. Cui et al. [64] overexpressed *aroAK* together with the feedback inhibition-resistant *aroG*^fbr^ from *E. coli*, which resulted in a twofold increase of MK-7 production compared to the wild type (Table 1).

We can also learn from studies on the production of aromatic chemicals and derivatives such as *p*-aminobenzoate, salicylate, *cis, cis*-muconic acid (MA), 4-hydroxycoumarin (4-HC), 4‑hydroxybenzoic acid, et cetera [65–68]. To inprove the microbial biosynthesis of 4-hydroxycoumarin, Lin et al. [69] constructed the chorismate-boosting plasmid pCS-APTA (overexpressing *aroL*, *ppsA*, *tktA* and *aroG*^fbr^), which led to the production of 283.9 mg/L 4HC, a 37% increase compared with its original strain. Additionally, strategies for the synthesis of chorismate derivatives are based on the release of pyruvate competition in many cases. So, utilization of metabolic engineering strategies involving the pyruvate recycling system combined with improved chorismate supply can promote cell growth, leading to high productivity and yield of chorismate derivatives. Cui et al. [64] designed a bifunctional Phr60-Rap60-Spo0A quorum-sensing molecular switch to fine-tune the expression of *pyk*, which limited the metabolic flux of pyruvate from PEP to the TCA cycle and enhanced its supply for MK-7 biosynthesis.

### Metabolic engineering of polyisoprene biosynthesis

**Fig. 3:**
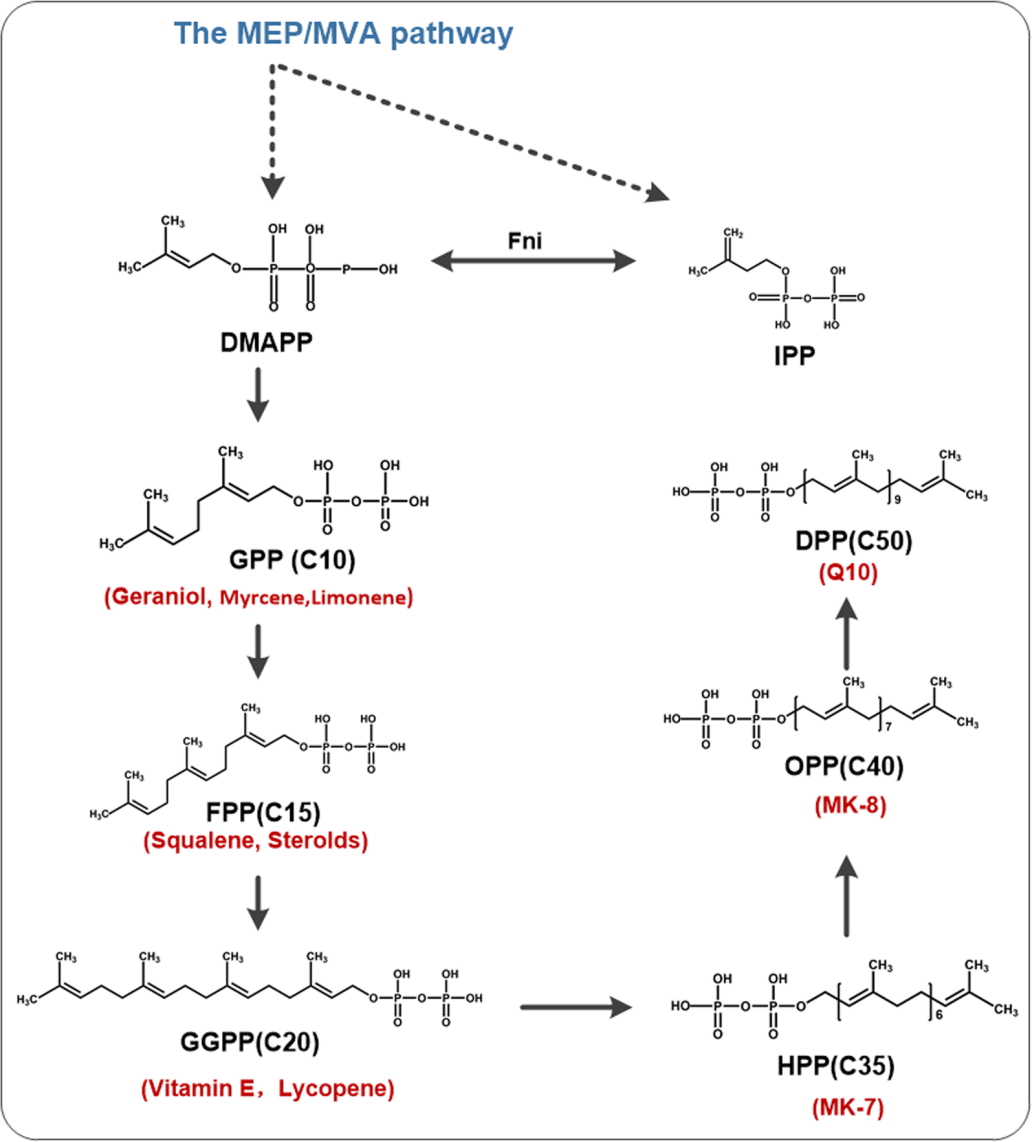
Isoprenoid‑derived molecules and the biosynthesis of isoprenoids, including the formation of C5 building blocks IPP and DMAPP by the MEP pathway and the MVA pathway. Red typeface indicates the spectrum of products derived from polyisoprene

More than 50,000 isoprenoid compounds are found in nature [70, 71]. Isoprenoids are a diverse group of molecules found in all organisms, where they perform a wide variety of important biological functions including electron transport, hormonal signaling, antioxidation, and growth regulation [70, 72–74]. The isoprenoid side chain anchors quinones in the lipid membrane, while the quinone head is responsible for the electron transfer capacity [41]. The long isoprenoid side chains, such as geranyl pyrophosphate (GPP), farnesyl pyrophosphate (FPP), geranylgeranyl pyrophosphate (GGPP), or heptaprenyl diphosphate (HPP) are synthesized by the consecutive condensation of the five-carbon monomer IPP and its isomer DMAPP (Fig. 3) [26].

Most bacteria biosynthesize the precursors of isoprenoids via the MEP pathway and the yield of isoprenoids can be increased by introducing a heterologous MVA pathway, which can function in parallel with the native MEP pathway to provide IPP and DMAPP [37]. Yuan et al. [44] knocked out *hepT* while simultaneously overexpressing *dxs*, *dxr*, and *ispD*–*ispF* in the MEP module, combine with the overexpression of the heterogeneous MVA module genes (*mvaK1, mvaK2, mvaD, mvaS, and mvaA*). This engineering strategy increased the MK-4 yield 11.1-fold to 90.1 ± 1.7 mg/L compared with the parental strain (Table 1) [44].

Pathway engineering of polyisoprene biosynthesis is also commonly used to improve the productivity of vitamin K2. The MEP pathway for the biosynthesis of IPP and DMAPP consists of eight enzyme-catalyzed reactions. Ma et al. [75] increased the titer of rate-limiting enzymes in the MEP pathway by the expressing the P*spac*-MenA-DxS-Dxr-Idi cassette, which resulted in an 11-fold increase of MK-7 production (Table 1). Yang et al. [63] improved the supply of heptaprenyl-PP by engineering the MEP pathway via overexpression of *dxs*, *dxr*, *ispD* (*yacM*), and *ispF* (*yacN*). However, overexpression of the other three pathway genes *ispE*, *ispH* (*yqfP*)*,* and *ispA* (*yqiD*) resulted in a decrease in the production of MK-7 without a clear explanations [63]. However, another study also optimized the MEP pathway to enhance MK-7 production. In this study, the *ispDFHG* genes were sequentially overexpressed in the BS20 strain, which intrinsically overexpresses *dxs* and *dxr*. The highest MK-7 titer of the resulting strain reached 415 ± 3.2 mg/L, representing a 29% increase over the original strain [76]. It is suggested that *ispE* overexpression might lead to an imbalance in the MEP pathway and the expression order of the genes had an impact on the MK-7 yield, which should be kept in mind when attempting to construct high-yield strains.

The MVA pathway does not exist in most prokaryotes, but a heterologous MVA pathway can be constructed in bacterial hosts. Gao et al. [77] developed an engineered *E. coli* strain for MK-7 production by introducing HepPPS (heptaprenyl pyrophosphate synthetase) from *B. subtilis* and optimizing the enzyme expression of the MVA pathway. This strategy increased the titer of MK-7 to 2.3 μM, which was 22-fold higher than that of the original strain (Table 1) [77].

Furthermore, the length of the isoprenoid side chain and the type of quinone differ among species [78]. For example, bacteria such as *B. subtilis* produce MK7, *E. coli* synthesize UQ8 and MK8, while yeasts such as *Saccharomyces cerevisiae* and *Schizosaccharomyces pombe* produce UQ6 and UQ10, respectively [41]. Notably, the isoprenoid pathway can be expanded to produce more diverse compounds such as limonene, lycopene, or vitamin E, and related studies offer great inspiration for the engineering of microbial factories for vitamin K2 production (Fig. 3) [38, 41, 79–83].

Additionally, increasing the precursor pools of pyruvate and G3P or acetyl-CoA is an important strategy for enhancing the synthesis of polyisoprenes. Yang et al. overexpressed *glpD* from the glycerol metabolism pathway in *B. subtilis* 168, which increased the yield of MK-7 to 13.7 ± 0.2 mg/L [63]. In the case of lycopene, which is also a terpenoid like vitamin K2, Farmer et al. guided the carbon flow from pyruvate to glycolysis back to G3P by overexpressing *pps* and deleting *pyk* in *E. coli* [84]. In order to increase the carbon flux of isoprene in *E. coli*, Kim et al. knocked out nine genes related to byproduct formation (*ackA-pta, poxB, ldhA, dld, adhE, pps,* and *atoDA*) to obtain the strain ApsPtM, which produced 1832 mg/L of isoprene [85]. The various compounds that have a polyisoprene structure similar to MK-7 are listed as examples for future research on the construction of vitamin K2 producing microbial factories.

### Metabolic engineering of MK biosynthesis

In *B. subtilis*, the biosynthesis of MK-7 proceeds via nine enzymatic reactions. The first six enzymes are encoded by the *menFDHBEC* operon and the *menG* gene is part of the *hepS-menG-hepT* operon, in which *hepS/hepT* encode all-trans heptaprenyl diphosphate synthase in the MEP pathway, while *menA* and *menI* are at separate loci in the genome [63]. Yang et al. [63] overexpressed each of the above cistrons using strong promoters in *B. subtilis* 168, and the results revealed that only the step reaction by MenA, i.e. the prenylation of DHNA to DMK was a rate-limiting step in the MK-7 pathway. Cui et al. [64] boosted the synthesis of MK-7 precursors in *B. subtilis* by overexpressing *menFBE*, but the results indicated that the encoded enzymes do not catalyze a rate-limiting step of MK-7 biosynthesis, which was consistent with Yang’s study mentioned before. Increasing the copy number of *menA* at a different locus increased the titer of MK-7 by 42 mg/L compared with the original strain [64]. These results show that *MenA* is a critical enzyme that can determine the efficiency of the MK-7 synthesis pathway.

Although the futalosine-dependent pathway is widely investigated as a target for the development of new herbicides and antibiotics, there have been no studies on the metabolic engineering of the futalosine pathway to increase the yield of menaquinone [26, 29]. It may be a promising strategy for the biosynthesis of vitamin K2 to combine these two MK biosynthesis pathways.

### Vitamin K2 and membrane engineering

The bilayer membrane is not only an interface between cell and the outside environment, but also contains intricate mechanism for signal transduction, transporters, cellular communication, adhesion, etc. [86, 87]. MK isoforms are lipid-soluble molecules that insert themselves into the bacterial cell membrane and shuttle electrons between the membrane-bound protein complexes in the electron-transport chain in many bacteria [41]. Hence, the synthesis of vitamin K2 may also affect by the state of the cell membrane, which has inspired studies of membrane engineering as a new direction for enhancing vitamin K2 biosynthesis.

Wang et al. [2] found that the stable surface tension and an optimized composition of the cell membrane are beneficial to the accumulation of MK-7. Furthermore, Cui et al. [88] showed that membrane components (GO:0006810, GO:0017000, GO:0008125, GO:0031224, GO:0051234) exhibited the most significantly changed expression levels in a comparative transcriptomic analysis of the intrinsic connections between biofilm formation and MK-7 production. Overexpression of cell-membrane-associated proteins such as signal receptors (BSU02010), transmembrane transporters (BSU29340, BSU03070), and *tatAD*-*CD* (BSU02630) could improve the synthesis of MK-7. These results directly indicate that the composition and homeostasis of the cell membrane can affect MK-7 synthesis. Co-expression of *tatAD*-*CD* (BSU02630) and menaquinol-cytochrome c reductase *qcrA*-*C* using the strong P43 promoter in the previously engineered strain BS20 increased the titer of MK-7 to 410 mg/L in shake-flask culture, which is the highest MK-7 titer reported to date [64, 88].

Similar to MK, lycopene, and carotenoids are biosynthesized from isoprene units and are accumulated in the cell membrane or neutral lipid droplets. Accordingly, there are reports that the yield of lycopene or carotenoids can be successfully improved by increasing the cellular lipid content (Fig. 3) [89, 90]. Coincidentally, Hu et al. [91] supplemented soybean oil to the medium, resulting in a maximal MK-7 yield of 40.96 mg/L. Therefore, expanding the product spectrum will be useful to promote the metabolic engineering design-build-test-learn cycle and possibly increase the economic viability of microbial cell factories.

## Conclusions and prospects

Traditionally, vitamin K is known for its essential role in the blood coagulation cascade and it is also involved in the maintenance of bone, preventing arterial hardening, modulating inflammation, and neuroprotection [9, 92]. Among vitamin K isoforms, MK-7 has gained widespread attention because its dietary intake offers numerous health benefits due to its longer half-life in human blood compared to other isoforms [15, 22]. With the progress of medical research, there is increasing evidence that the current recommended dietary supply of vitamin K2 cannot meet the needs of many individuals, and the market demand for vitamin K2 supplements is increasing significantly [9, 93–95].

Vitamin K2 plays a key role in the prokaryotic respiratory electron transport chain. Bacteria and archaea are the main producers of menaquinone [17, 96]. Different strains can produce different types of menaquinone. MK-7 is mainly produced by fermentation of *B. subtilis,* and its proportion among all isoforms can reach 90–96% [97]. Due to its long history of research and high vitamin K2 content, *B. subtilis* is considered the most promising potential strain for microbial production of MK-7. However, due to the long fermentation time and high industrial cost of *B. subtilis*, engineering *E. coli* to synthesize MK-7 is considered an attractive alternative for the economical industrial production of MK-7.

Chemicals derived from isoprenoids and chorismate are highly valuable and can be applied in various industrial areas. Although many successful case studies offer hope for the industrial biosynthesis of MK-7, the obtainable titer are still too low. The inherent constraints of the MK-7 microbial factory are related to the complex and strictly regulated pathways including 37 enzymes, multiple cofactors, and a series of reactions from glucose. Firstly, the coordination of the different metabolic pathways leads to tradeoffs in menaquinone biosynthesis. For example, pyruvate and PEP are important precursors for the synthesis of the isoprene side chain and chorismate, respectively. But most PEP is converted into pyruvate, which needs to enter the TCA cycle to supply ATP and NADH. Thus, balancing the fiuxes of PEP and pyruvate by designing dynamic regulatory is critical for the biosynthesis of different MK isoforms. The application of bifunctional pyruvate-responsive biosensor for dynamic dual control (activation and inhibition) to fine-tune the MK-7 yield is a promising approach for solving this problem [98]. In addition to coordinating complex pathways, it is also necessary to regulate the expression and activity of various enzymes, which also limits of vitamin K2 biosynthesis. Even if the same enzyme is expressed in chassis cells, the timing of expression and differences between chassis cells may have opposite effects on the yield of MK-7 (unpublished data). Another major bottleneck is the feedback inhibition of key enzymes, which also restricts the biosynthesis of vitamin K2. For example, DAHP synthase in the shikimate biosynthesis pathway is subject to feedback-inhibition by aromatic amino acids. The first committed enzyme of menaquinone biosynthesis in the human pathogen *Mycobacterium tuberculosis* (Mtb), MenD, is subject to feedback inhibition by direct binding of a downstream metabolite from the biosynthetic pathway, DHNA [99]. The allosteric binding sites of different bacterial MenD enzymes show limited conservation, and the regulation of the pathway by DHNA is limited to certain bacteria. Thus, feedback inhibition requires in-depth study for each newly discovered enzyme. Moreover, the rate-limiting enzymes, such as MenA, have low expression levels and the accumulation of HMBPP in the MEP pathway can significantly affected cell growth and isoprenoid production. Thus, balancing the metabolic flux of the biosynthetic enzymes is critical for vitamin K2 biosynthesis [75, 100].

The product spectrum related to vitamin K2 is a double-edged sword. Not only can it inspire metabolic engineering strategies of vitamin K2 biosynthesis, but it also means that related products in the cell compete for intermediate metabolites. For example, the (C5) isoprenoids DMAPP and IPP formed in either the MVA or the MEP pathway are condensed to form (C55) undecaprenyl diphosphate (UPP), which participates in cell wall biosynthesis. Therefore, the biosynthesis of UPP can compete with vitamin K2 synthesis for IPP and DMAPP, but blocking this process is bound to have a serious impact on cell growth [101]. Nevertheless, we can still learn from successful metabolic engineering strategies such as pathway engineering and membrane engineering from the product spectrum. In the past decade, the MEP pathway together with the MVA pathway and the shikimate route were engineered in microorganisms to produce exciting yields of downstream products. With the development of microbial biotechnology, MK-7 will become available at high production yields and low cost in the future.

Essentially, this review aims to provide an overview of the strategies for the microbial production of vitamin K2 based on the engineering of the shikimate pathway, polyisoprene biosynthesis and MK pathway. More importantly, we expand the spectrum of products similar to vitamin K2, which gives us more insights into available strain engineering strategies. Additionally, the intrinsic characteristics of the strain such as biofilm formation, cell membrane composition and electron transfer chain can provide inspiration for strategies that can enhance the production of MK-7. The utilization of more advanced biotechnological methods, such as dynamic regulation, computer-aided design of metabolic networks, and metabolic flux balance analysis will further increase the productivity and reduce the cost of microbial vitamin K2 fermentation to ultimately realize its industrial production.

## Data Availability

Not applicable.

## Enzymes

GlpK: Glycerol kinase
GlpD: Glycerol-3-phosphate dehydrogenase
TpiA: Triosephosphate isomerase
Tkt: Transketolase
PykA: Pyruvate kinase II
AroA: 3-Deoxy-7-phosphoheptulonate synthase
AroB: 3-Dehydroquinate synthase
AroC: 3-Dehydroquinate dehydratase I
AroD: Shikimate dehydrogenase
AroK: Shikimate kinase
AroE: 3-Phosphoshikimate 1-carboxyvinyltransferase
AroF: Chorismate synthase
AtoB: Acetyl-CoA C-acetyltransferase
Erg10: Acetyl-CoA acetyl transferase
Erg13: Hydroxymethylglutaryl-CoA synthase
Hmg1/Hmg2: Hydroxymethylglutaryl-CoA reductase
Erg12: Mevalonate kinase
Erg8: Phosphomevalonate kinase
Erg19: Mevalonate pyrophosphate decarboxylase
DxS: 1-Deoxyxylulose-5-phosphate synthase
DxR: 1-Deoxyxylulose-5-phosphate reductoisomerase
IspD: 2-C-methylerythritol 4-phosphate cytidylyltransferase
IspE: 4-Diphosphocytidyl-2-C-methylerythritol kinase
IspF: 2-C-methylerythritol 2,4-cyclodiphosphate synthase
IspG: 4-Hydroxy-3-methylbut-2-enyl diphosphate synthase
IspH: 4-Hydroxy-3-methylbut-2-enyl diphosphate reductase
Idi: Isopentenyl-diphosphate delta-isomerase
IspA: Farnesyl diphosphate synthase
HepS/T: Heptaprenyl diphosphate synthase
MenF: Isochorismate synthase
MenD: 2-Succinyl-5-enolpyruvyl-6-hydroxy-3-cyclohexene-1-carboxylate synthase
MenH: Demethylmenaquinone methyltransferase
MenC: *O*-Succinylbenzoate synthase
MenE: *O*-Succinylbenzoate-CoA ligase
MenB: 1,4-Dihydroxy-2-naphthoyl-CoA synthase
MenI: 1,4-Dihydroxy-2-naphthoyl-CoA hydrolase
MenA: 1,4-Dihydroxy-2-naphthoate heptaprenyltransferase
MenG: Demethylmenaquinone methyltransferase
MqnA: Chorismate dehydratase
MqnE: Aminodeoxyfutalosine synthase
MqnF: Aminodeoxyfutalosine deaminase
MqnB: Futalosine hydrolase
MqnC: Cyclic dehypoxanthinyl futalosine synthase
MqnD: 1,4-Dihydroxy-6-naphthoate synthase
MTAN: Adenosylhomocysteine/aminodeoxyfutalosine nucleosidase
MvaE: Acetyl-CoA acetyltransferase/HMG-CoA reductase
MvaS: Hydroxymethylglutaryl-CoA synthase
MVK: Mevalonate kinase
UbiC: Chorismate lyase
UbiA: 4-Hydroxybenzoate octaprenyltransferase
HepPPS: Heptaprenyl pyrophosphate synthetase
mvaK1: Mevalonate kinase
mvaK2: Phosphomevalonate kinase
mvaD: Diphosphomevalonate decarboxylase
mvaA: Acetyl-CoA acetyltransferase
hsUBIAD1: *Homo sapiens* Geranylgeranyl diphosphate synthase

## Metabolites

Glu: Glucose
F6P: d-Fructose 6-phosphate
DAHP: Dihydroxyacetone phosphate
GAP: d-Glyceraldehyde 3-phosphate
E4P: Erythrose 4-phosphate
PEP: Phosphoenolpyruvate
PYR: Pyruvate
TCA cycle: Citrate cycle
CHA: Chorismate
DHAP: 3-Deoxy-darobino-heptulosonate 7-phosphate
DHQ: 3-Dehydroquinate
SA: Shikimate
DHS: 3-Dehydroshikimate
S3P: Shikimate 3-phosphate
EPSP: 5-Enolpyruvoylshikimate 3-phosphate
AcCoA: Acetyl-CoA
AACoA: Acetoacetyl-CoA
HMG-CoA: 3-Hydroxy-3-methyl-glutaryl-CoA
MVA: Mevalonate
MVAP: Mevalonate-5P
MVAPP: Mevalonate-5PP
DXP: 1-Deoxy-d-xylulose 5-phosphate
MEP: 2-C-methyl-d-erythritol 4-phosphate
CDP-ME: 4-(Cytidine 5′-diphospho)-2-C-methyl-d-erythritol
CDP-MEP: 2-Phospho-4-(cytidine 5′-diphospho)-2-C-methyl-d-erythritol
MEcPP: 2-C-methyl-d-erythritol 2,4-cyclodiphosphate
HMBPP: 1-Hydroxy-2-methyl-2-butenyl 4-diphosphate
DMAPP: Dimethylallyl diphosphate
IPP: Isopentenyl diphosphate
GPP: Geranyl diphosphate
FPP: Farnesyl diphosphate
GGPP: Geranylgeranyl diphosphate
HPP: All-*trans*-Heptaprenyl diphosphate
ISoCHA: Isochorismate
SEPHCHC: 2-Succinyl-5-enolpyruvyl-6-hydroxy-3-cyclohexene-1-carboxylate
SHCHC: (1R,6R)-2-succinyl-6-hydroxy-2,4-cyclohexadiene-1-carboxylate
DHNA: 1,4-Dihydroxy-2-naphthoate
DMK-7: Demethylmenaqunol-7
OPP: All-*trans*-octaprenyl diphosphate
DPP: All-*trans*-decaprenyl diphosphate
MK: Menaquinone
